# MRI-based biomarkers of bone marrow adaptation in anemia: A quantitative evaluation

**DOI:** 10.1007/s00256-026-05251-x

**Published:** 2026-05-18

**Authors:** Duygu Erkal, Mehmet Tonkaz, Serdar Aslan, Gökhan Tonkaz

**Affiliations:** Department of Radiology, Faculty of Medicine, Giresun University, Giresun, Turkey

**Keywords:** Anemia, Bone marrow, Quantitative MRI, Fat fraction, ADC, RDW, Red marrow hyperplasia

## Abstract

**Objective:**

To evaluate anaemia-related alterations in lumbar bone marrow using apparent diffusion coefficient (ADC), fat fraction (FF), and signal intensity ratio (SIO), and to assess relationships with haematologic markers.

**Methods:**

Ninety-three patients with abdominal MRI and available haemoglobin (Hb), red cell distribution width–coefficient of variation (RDW-CV), red cell distribution width–standard deviation (RDW-SD), and quantitative MRI parameters were included. Patients were classified into four Hb-based groups. Group differences were assessed using Kruskal–Wallis and Dunn–Bonferroni tests. Spearman correlation, age- and BMI-adjusted partial correlations and intraclass correlation coefficients (ICCs) were computed.

**Results:**

Hb showed a positive correlation with FF (rho = 0.386, *p* < 0.001) and a negative correlation with ADC (rho =  − 0.467, *p* < 0.001). RDW-CV correlated negatively with FF (rho =  − 0.341, *p* = 0.001) and positively with ADC (rho = 0.300, *p* = 0.004). RDW-SD showed a weak positive correlation with ADC (rho = 0.208, *p* = 0.045). After age adjustment, RDW-SD correlated with FF (partial rho =  − 0.350, *p* = 0.001). The initially borderline RDW-SD–ADC association became significant after age (partial rho = 0.279, *p* = 0.007) and BMI adjustment (partial rho = 0.219, *p* = 0.035). FF and ADC differed across groups (*p* < 0.05). FF was lowest in Group 4 (all *p* < 0.05). ADC was higher in Group 4 than in Groups 1 and 2 (*p* ≤ 0.001) and higher in Group 3 than Group 1 (*p* = 0.035). All ICC values exceeded 0.90.

**Conclusion:**

Quantitative MRI parameters reflect anaemia-related lumbar bone marrow microstructural alterations.

## Introduction

Anaemia is a common haematologic disorder characterized by reduced tissue oxygen-carrying capacity resulting from decreased haemoglobin (Hb) concentration in the blood. It may arise from a broad spectrum of etiologic factors, including iron deficiency, chronic diseases, blood loss, bone marrow failure, or haemolytic processes [1–3].

Sex and age-related differences are important parameters when assessing anaemia. According to the World Health Organization (WHO) criteria [1], threshold values defining anaemia are presented in Table 1. This classification serves as a fundamental reference for distinguishing the severity of haematologic alterations and their corresponding effects at the tissue level.

**Table 1:** Anemia classification based on hemoglobin concentration (g/L) according to age, sex, and pregnancy status

Group	No Anemia	Mild Anemia	Moderate Anemia	Severe Anemia
Children, 6–23 months	≥ 105	95–104	70–94	< 70
Children, 24–59 months	≥ 110	100–109	70–99	< 70
Children 5–11 years	≥ 115	110–114	80–109	< 80
Children, 12–14 years (girl)	≥ 120	110–119	80–109	< 80
Children, 12–14 years (boy)	≥ 120	110–119	80–109	< 80
Adults, 15–65 years (women)	≥ 120	110–119	80–109	< 80
Adults, 15–65 years (men)	≥ 130	110–129	80–109	< 80
Pregnancy – 1 st trimester	≥ 110	100–109	70–99	< 70
Pregnancy – 2nd trimester	≥ 105	95–104	70–94	< 70
Pregnancy – 3rd trimester	≥ 110	100–109	70–99	< 70

The clinical effects of anaemia extend beyond the reduction in oxygen-carrying capacity. Systemic metabolic adaptations that develop secondary to hypoxia lead to marked alterations in cellular energy metabolism, oxidative stress responses, and the integrity of tissue microstructure. These adaptive processes trigger remodelling within hematopoietic tissue and result in changes in the cellular composition and fat-water balance of the bone marrow stroma. Increased erythropoietic activity in response to hypoxia reduces stromal adipocytes and promotes the restructuring of hematopoietic tissue, producing measurable morphological and biochemical changes in bone marrow architecture [2, 3].

The ability to noninvasively evaluate these microstructural changes has become possible with advanced quantitative magnetic resonance imaging (MRI) techniques. Diffusion-weighted imaging (DWI) provides insight into cellular density and microcirculation by reflecting the Brownian motion of water molecules within tissues. The apparent diffusion coefficient (ADC), derived from DWI, serves as an indirect biomarker of diffusion restriction and tissue cellularity [4]. In addition, the fat fraction (FF) obtained from chemical-shift-based Dixon sequences enables quantitative assessment of tissue lipid content [5, 6], while the in-phase/out-phase ratio (SIO) measures the phase difference between fat and water protons, offering complementary information on the degree of tissue fat content. These MRI-derived parameters are particularly relevant in anaemia, as increased haematopoietic demand leads to reconversion from fatty (yellow) marrow to haematopoietically active (red) marrow, resulting in reduced marrow fat content and associated microstructural alterations. These compositional changes can be quantitatively detected using MRI-derived parameters such as FF, ADC, and SIO. The combined use of these parameters allows a more comprehensive evaluation of bone marrow composition [6].

Recent studies have shown that MRI-based quantitative parameters may demonstrate meaningful associations with hematologic indicators. Tsujikawa et al. reported that lumbar vertebral ADC correlated significantly with age and red cell distribution width (RDW), and additionally that iliac ADC was positively associated with Hb levels [7]. Chen et al. demonstrated that pelvic bone marrow DWI signal intensity increased proportionally with the severity of anaemia, whereas changes in ADC remained limited [8]. Similarly, analyses in patients with aplastic anaemia and multiple myeloma have indicated that increases in fat fraction reflect the loss of hematopoietic cells [9, 10].

These findings support the feasibility of evaluating the tissue-level effects of anaemia using noninvasive imaging methods. However, most existing studies focus on assessing bone marrow microstructure in specific patient groups, such as those with hematologic malignancies, aplastic anaemia, or multiple myeloma [11–15], and very few studies have systematically investigated anaemia-related marrow changes in non-malignant populations. Furthermore, prior research has typically evaluated ADC, FF, or SIO in isolation, rather than in combination, limiting the ability to determine which parameter is most sensitive to varying degrees of anaemia [16–20]. To date, no comprehensive study has systematically compared lumbar vertebral ADC, FF, and SIO parameters together across different anaemia severity categories.

The aim of this study was to evaluate lumbar vertebral bone marrow alterations using quantitative MRI parameters (ADC, FF, and SIO) and to assess their relationships with haematologic indices across different anaemia severity groups.

## Material-methods

### Study population

This retrospective, single-centre study was conducted by selecting eligible cases among abdominal MRI examinations performed in the radiology department of our institution between January 2024 and June 2025. Ethical approval for the study was obtained from the institutional review board.

Patients were included if their MRI examinations encompassed the lumbar vertebral levels and if Hb (g/dl) and Red Cell Distribution Width-Coefficient of Variation (RDW-CV, %) and Red Cell Distribution Width-Standard Deviation (RDW-SD, fL) levels had been measured within one week before or after the imaging date. Patients with a history of hematologic malignancy, those with bone metastases, prior chemotherapy or radiotherapy, or examinations deemed to have insufficient image quality due to motion artifacts were excluded. Based on these criteria, a total of 93 patients were included in the final analysis.

Patients were categorized into four groups based on sex-specific WHO haemoglobin thresholds (Table 2): Group 1 (normal) (*n* = 26; 15 male, 11 female), Group 2 (mild anaemia) (*n* = 24; 13 male, 11 female), Group 3 (moderate anaemia) (*n* = 25; 11 male, 14 female), and Group 4 (severe anaemia) (*n* = 18; 10 male, 8 female). All participants were non-pregnant adults, and anaemia severity was classified according to WHO criteria for non-pregnant individuals. The demographic and MRI characteristics of these groups are summarized in Table 2.

**Table 2:** Overview of median (IQR) demographic data and lumbar bone marrow MRI metrics across sex-specific WHO hemoglobin–defined anemia groups

Parameter	Group 1 (Normal, *n* = 26)	Group 2 (Mild Anemia, *n* = 24)	Group 3 (Moderate Anemia, *n* = 25)	Group 4 (Severe Anemia, *n* = 18)
Age	62.5 (30.75–64)	56.0 (41.75–63.75)	60.0 (53.0–65.0)	54.5 (40.0–63)
Gender	15 male (57.7%), 11 female (42.3%)	13 male (54.2%), 11 female (45.8%)	11 male (44.0%), 14 female (56.0%)	10 male (55.6%), 8 female (44.4%)
Hb (g/dl)	12.55 (12.2–13.23)	11.8 (11.6–11.8)	10.8 (10.5–10.9)	7.45 (7–7.8)
RDW-CV (%)	14.25 (13.05–16.5)	14.2 (13.32–15.75)	14.8 (13.55–15.65)	19.35 (17.32–22.98)
RDW-SD (fL)	43.95 (40.28–46.66)	43.35 (40.33–47.5)	44.2 (41.5–48.45)	50.85 (43.78–61)
FF (%)	37 (29.25–40)	33.5 (27–37.75)	35 (23–38)	22 (15–27.75)
ADC (10^−6^ mm^2^/s)	389.5 (346.25–428.25)	410.5 (371.5–446)	460 (358.5–527)	502 (442.50–579)
SIO	0.32 (0.20–0.52)	0.29 (0.2–0.35)	0.28 (0.20–0.33)	0.27 (0.16–0.43)

The etiologies of anaemia were obtained from clinical records and included iron deficiency anaemia, anaemia of chronic disease, haemolytic anaemia, and vitamin B12 deficiency. In the mild anaemia group, the most frequent cause was iron deficiency anaemia (*n* = 18), followed by anaemia of chronic disease (*n* = 4) and haemolytic anaemia (*n* = 2). In the moderate anaemia group, etiologies included iron deficiency anaemia (*n* = 10), anaemia of chronic disease (*n* = 10), haemolytic anaemia (*n* = 3), and vitamin B12 deficiency (*n* = 2). In the severe anaemia group, anaemia of chronic disease (*n* = 14) predominated, followed by iron deficiency anaemia (*n* = 4).

### MRI technique and image analysis

All abdominal MRI examinations were performed on a 1.5 T system (Aera, Siemens Healthineers, Erlangen, Germany) using a 16-channel phased-array body coil. The field of view included the upper abdomen and lumbar vertebral bodies.

Diffusion-weighted imaging (DWI) was acquired using a single-shot echo-planar imaging (ss-EPI) sequence with *b*-values of 50, 400, and 800 s/mm^2^ to generate ADC maps. Chemical-shift-based Dixon imaging was used to obtain in-phase (IP) and out-of-phase (OP) images for calculating the signal intensity ratio (SIO) and to produce proton density fat fraction (PDFF) maps. PDFF maps were generated using a multi-echo qDixon acquisition with T2*-corrected fat fraction estimation. Key acquisition parameters were as follows: T1W VIBE Dixon: TR 7.1 ms, TE 2.39/4.77 ms, flip angle 10°, FOV 400 mm, matrix 320 × 144, NEX 2, slice thickness 4 mm. qDixon: TR 7.35 ms, TEs 2.38–14.28 ms (ΔTE 2.38 ms), flip angle 4°, FOV 400 mm, matrix 320 × 144, NEX 1, slice thickness 2 mm. DWI: TR 1700 ms, TE 73 ms, flip angle 90°, FOV 380 mm, matrix 320 × 144, NEX 2, slice thickness 6 mm.

IP/OP images, PDFF maps, and ADC maps were reviewed for quantitative analysis using Syngo.via software (Siemens Healthineers, Erlangen, Germany). Measurement locations were determined by consensus between two radiologists, each with 8 years of experience. All quantitative measurements were subsequently performed by one of these radiologists for consistency. To assess inter-reader reliability, a third radiologist with 10 years of experience independently defined the ROI locations and performed all measurements, blinded to the first reader’s measurements and to clinical and laboratory data. For intra-reader reliability, the same radiologist who performed the initial measurements repeated them after a minimum interval of 12 weeks.

**Fig. 1:**
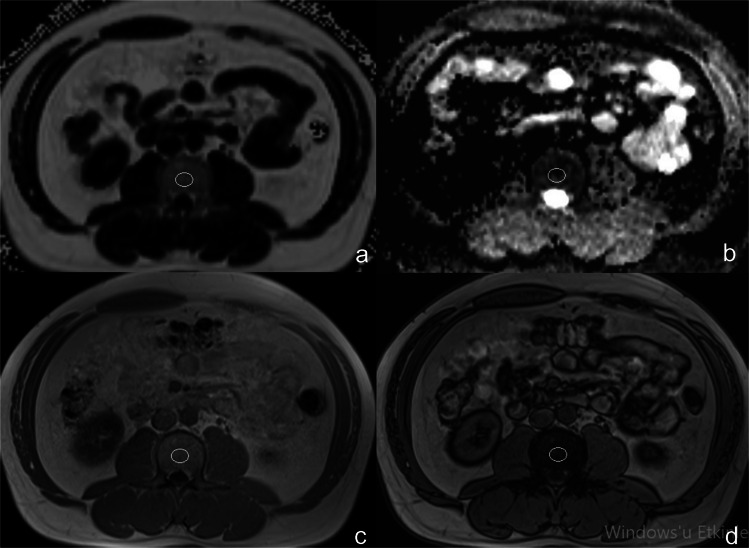
45-year-old male patient from the Group 1. Lumbar measurements obtained at L2 level on PDFF (**a**), ADC (**b**), in-phase (**c**), and out-of-phase (**d**) images showed vertebral FF: 46%, ADC: 335 × 10⁻^6^ mm^2^/s, and SIO: 0.35. Laboratory results demonstrated Hb:14.6 g/dL, RDW-CV: 12.8%, and RDW-SD: 40.2 Fl

**Fig. 2:**
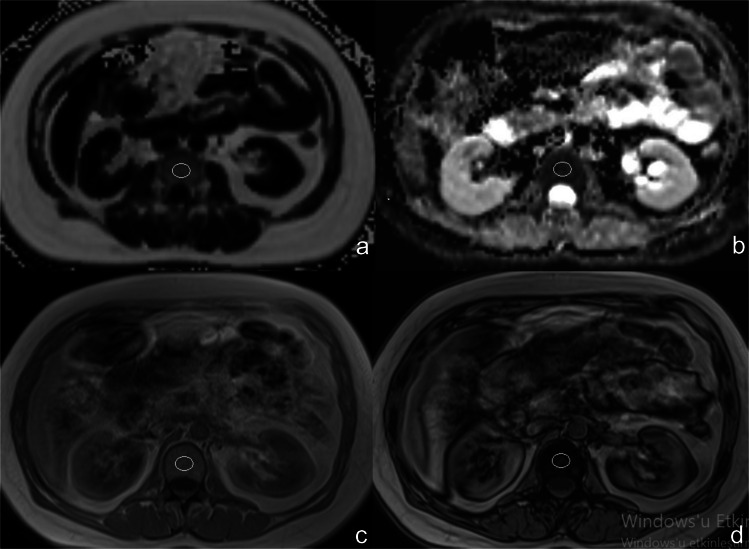
48-year-old female patient from the Group 3. Lumbar measurements obtained at L2 level on PDFF (**a**), ADC (**b**), in-phase (**c**), and out-of-phase (**d**) images showed vertebral FF: 35%, ADC: 480 × 10⁻^6^ mm^2^/s, and SIO: 0.28. Laboratory results demonstrated Hb:10 g/dL, RDW-CV: 15.8%, and RDW-SD: 45.3 Fl

**Fig. 3:**
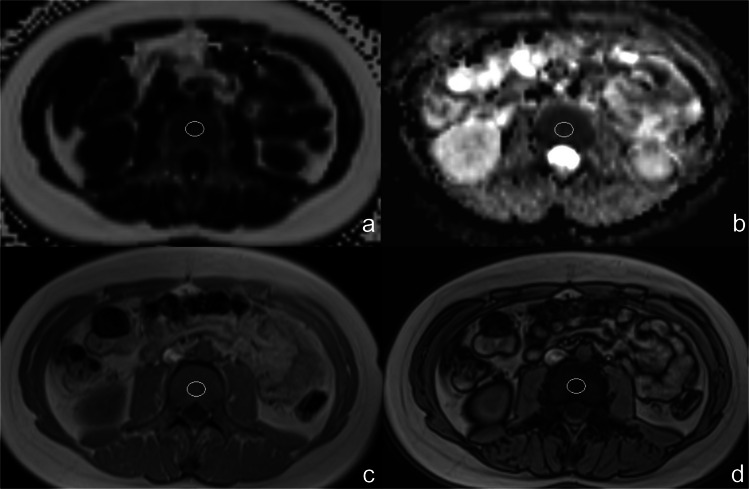
19-year-old female patient from Group 4. Lumbar measurements obtained at L2 level on PDFF (**a**), ADC (**b**), in-phase (**c**), and out-of-phase (**d**) images showed vertebral FF: 15%, ADC: 588 × 10⁻^6^ mm^2^/s, and SIO: 0.45. Laboratory results demonstrated Hb:7.5 g/dL, RDW-CV: 24.8%, and RDW-SD: 59.3 fL

Although the abdominal MRI protocol included multiplanar acquisitions, all quantitative measurements in this study were performed exclusively on axial images to ensure methodological consistency and reproducibility. Regions of interest (ROIs) were manually placed within the L2 vertebral body on axial images, avoiding cortical bone, endplates, vascular structures, and focal lesions. If the L2 vertebra contained a benign lesion (e.g., hemangioma) or other findings that could interfere with reliable measurement, the L3 vertebral body was used instead. ROI size was standardized to an approximate range of 1–1.5 cm^2^, with minor adjustments allowed to accommodate interpatient variability in vertebral morphology and to avoid partial-volume contamination from cortical bone and vascular channels. From each ROI, the following quantitative parameters were recorded: ADC (10⁻^6^ mm^2^/s), fat fraction (FF, %), and signal intensity ratio (SIO = OP/IP). Figures 1, 2, 3 show example cases from the study population.

### Statistical analysis

A priori power analysis was performed using G*Power (version 3.1) to determine the required sample size for detecting a moderate correlation (*r* = 0.30) with a two-tailed α = 0.05 and a statistical power of 0.80.

All statistical analyses were performed by the corresponding author using SPSS software (v.26.0; IBM Corp., Armonk, NY, USA) and R software (v.4.5.3; R Foundation for Statistical Computing, Vienna, Austria). The distribution of continuous variables was assessed using the Shapiro–Wilk test. Normally distributed data were summarized as mean ± standard deviation, whereas non-normally distributed variables were expressed as median and interquartile range (IQR).

Associations between hematologic markers (Hb, RDW-CV, RDW-SD) and MRI-derived parameters (FF, ADC, SIO) were examined using Spearman’s rank correlation analysis. Spearman’s rank correlation was used instead of Pearson correlation, as several variables did not meet the assumption of normality based on the Shapiro–Wilk test, and Spearman’s method is more appropriate for non-normally distributed data and monotonic relationships. Additionally, partial Spearman correlation analyses were performed with adjustment for age and BMI to account for potential confounding effects. To obtain more robust confidence intervals (CIs), bootstrapped 95% CIs were computed using the bias-corrected and accelerated (BCa) method with 2000 resamples for all Spearman and partial Spearman correlation analyses.

Group comparisons of FF, ADC, and SIO across the four anemia categories (normal, mild, moderate, severe) were performed using the Kruskal–Wallis test, followed by the Dunn–Bonferroni post hoc test for pairwise comparisons when the Kruskal–Wallis test indicated a statistically significant difference. Age and BMI differences among the anemia groups were also evaluated using the Kruskal–Wallis test. Sex distribution across the anemia groups was assessed using the chi-square (χ2) test**.** A *p*-value < 0.05 was considered statistically significant.

Intraclass correlation coefficients (ICCs) with 95% CIs were calculated using a two-way random-effects model with absolute agreement to assess inter- and intra-reader reliability. ICC values were interpreted as follows: < 0.50 poor, 0.50–0.75 moderate, 0.75–0.90 good, and > 0.90 excellent agreement [21].

## Results

Power analysis indicated that a minimum of 85 participants was required. Our final sample of 93 patients exceeded this threshold, confirming adequate statistical power for correlation analyses. A total of 93 patients were classified into four anaemia groups based on hemoglobin levels. The median values of age, Hb, RDW indices, FF, ADC, and SIO for each group are summarized in Table 2. Age and BMI distribution were similar across the anaemia groups, with no significant difference observed (*p* = 0.508 and *p* = 0.909, respectively). Likewise, sex distribution did not differ significantly among the four groups (χ^2^ = 1.10, *p* = 0.777).

**Fig. 4:**
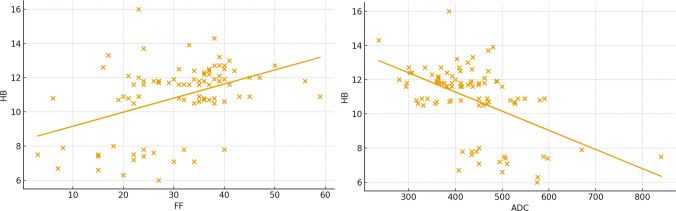
Scatter plots illustrating the Spearman correlation between hemoglobin (Hb, g/dL) and fat fraction (FF, %) (**a**), and between Hb and apparent diffusion coefficient (ADC, × 10⁻⁶ mm^2^/s) (**b**). The regression line represents the trend of association. Data are presented for all participants (*n* = 93)

Spearman correlation analysis revealed a moderate positive correlation between Hb and FF (rho = 0.386, 95% CI: 0.191 to 0.555, *p* < 0.001) and a moderate negative correlation between Hb and ADC (rho = –0.467, 95% CI: –0.616 to –0.290, *p* < 0.001). RDW-CV correlated negatively with FF (rho = –0.341, 95% CI: –0.510 to –0.153, *p* = 0.001) and positively with ADC (rho = 0.300, 95% CI: 0.098 to 0.478, *p* = 0.004). RDW-SD showed a weak positive correlation with ADC (rho = 0.208, 95% CI: –0.005 to 0.403, *p* = 0.045), whereas its association with FF did not reach significance in unadjusted analysis (rho = –0.187, 95% CI: –0.370 to 0.031, *p* = 0.072). No significant associations were observed for SIO. After adjusting for age, all significant correlations persisted and RDW-SD additionally demonstrated a significant negative correlation with FF (partial rho = –0.350, 95% CI: –0.503 to –0.133, *p* = 0.001). Moreover, the initially borderline RDW-SD–ADC association became statistically significant after both age (partial rho = 0.279, 95% CI: 0.075 to 0.462, *p* = 0.007) and BMI adjustment (partial rho = 0.219, 95% CI: 0.013 to 0.410, *p* = 0.035). BMI-adjusted analyses yielded largely consistent results. Full details are provided in Table 3 and Fig. 4.

**Table 3:** Spearman and partial Spearman correlation coefficients (age- and BMI-adjusted) with bootstrapped 95% confidence intervals (CIs) between hematologic indices (Hb, RDW-CV, RDW-SD) and quantitative MRI parameters (FF, ADC, SIO)

Hematologic index	MRI parameter	Spearman rho (95% CI)	*p*-value	Partial Spearman rho, age-adjusted(95% CI)	*p*-value	Partial Spearman rho, BMI-adjusted (95% CI)	*p*-value
Hb	**FF**	**0.386 (0.191, 0.555)**	** < 0.001**	**0.435 (0.256, 0.574)**	** < 0.001**	**0.387 (0.186, 0.556)**	** < 0.001**
Hb	**ADC**	** − 0.467 (− 0.616, − 0.290)**	** < 0.001**	** − 0.477 (− 0.619, − 0.303)**	** < 0.001**	** − 0.468 (− 0.622, − 0.297)**	** < 0.001**
Hb	SIO	0.116 (− 0.110, 0.327)	0.255	0.114 (− 0.113, 0.331)	0.267	0.116 (− 0.111, 0.324)	0.270
RDW-CV	**FF**	** − 0.341 (− 0.510, − 0.153)**	**0.001**	** − 0.408 (− 0.554, − 0.235)**	** < 0.001**	** − 0.345 (− 0.508, − 0.148)**	** < 0.001**
RDW-CV	**ADC**	**0.300 (0.098, 0.478)**	**0.004**	**0.315 (0.114, 0.496)**	**0.002**	**0.304 (0.100, 0.483)**	**0.003**
RDW-CV	SIO	− 0.035 (− 0.261, 0.208)	0.742	− 0.036 (− 0.260, 0.205)	0.735	− 0.041 (− 0.265, 0.189)	0.698
RDW-SD	**FF**	− 0.187 (− 0.370, 0.030)	0.072	** − 0.350 (− 0.503, −0.133)**	**0.001**	− 0.197 (− 0.386, 0.020)	0.059
RDW-SD	**ADC**	**0.208 (− 0.005, 0.403)**	**0.045**	**0.279 (0.075, 0.462)**	**0.007**	**0.219 (0.013, 0.410)**	**0.035**
RDW-SD	SIO	− 0.021 (− 0.242, 0.214)	0.839	− 0.039 (− 0.254, 0.186)	0.716	− 0.042 (− 0.266, 0.186)	0.689

**Fig. 5:**
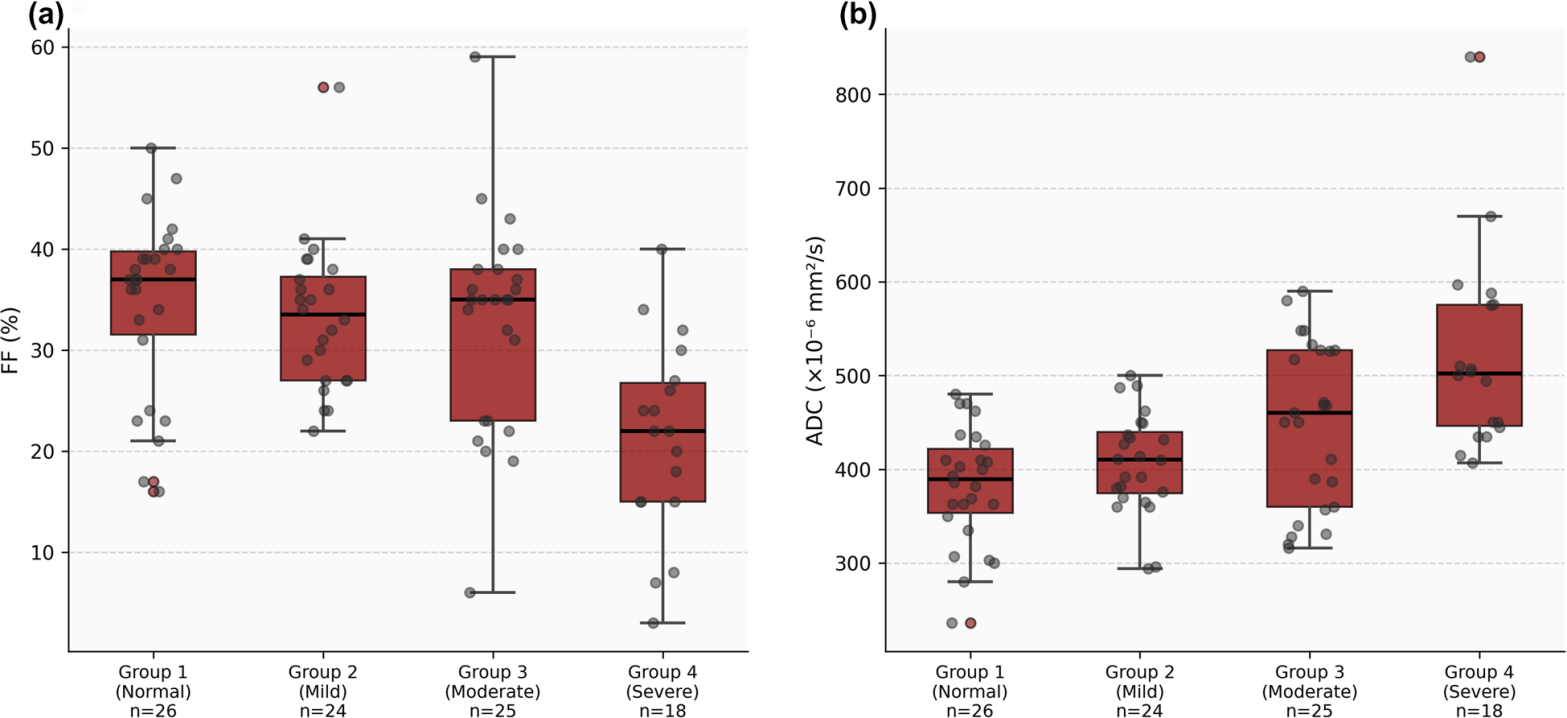
Box plots showing the distribution of (**a**) fat fraction (FF, %) and (**b**) apparent diffusion coefficient (ADC, × 10⁻⁶ mm^2^/s) across the four anemia severity groups. Individual data points are overlaid

In comparisons among the four anaemia groups, FF values showed statistically significant differences (*p* < 0.001). Post hoc analyses demonstrated that Group 4 had significantly lower FF values than all other groups (Group 1 vs Group 4: *p* < 0.001; Group 2 vs Group 4: *p* = 0.005; Group 3 vs Group 4: *p* = 0.006)**.** Similarly, ADC values also differed significantly between groups (*p* < 0.001). Group 4 exhibited significantly higher ADC values compared with Group 1 (*p* < 0.001) and Group 2 (*p* = 0.001). In addition, Group 3 showed higher ADC values than Group 1 (*p* = 0.035). No significant differences were observed in SIO values across the groups (Table 4, Fig. 5).

**Table 4:** Kruskal–Wallis and Dunn–Bonferroni post hoc test results for FF, ADC, and SIO across the four anemia groups (Group 1: normal, Group 2: mild anemia, Group 3: moderate anemia, and Group 4: severe anemia)

Parameter	*p*-value	Group 1-Group 2	Group 1-Group 3	Group 1-Group 4	Group 2-Group 3	Group 2-Group 4	Group 3-Group 4
FF	** < 0.001**	1.000	1.000	** < 0.001**	1.000	**0.005**	**0.006**
ADC	** < 0.001**	1.000	**0.035**	** < 0.001**	0.531	**0.001**	0.185
SIO	0.763	-	-	-	-	-	-

Inter-reader agreement for ADC, FF, and SIO measurements was excellent, with ICC values of 0.940 (95% CI: 0.911–0.960), 0.939 (95% CI: 0.909–0.959), and 0.945 (95% CI: 0.908–0.959), respectively. Intra-reader agreement also demonstrated excellent repeatability, with ICC values of 0.957 (95% CI: 0.936–0.967) for ADC, 0.921 (95% CI: 0.882–0.946) for FF, and 0.949 (95% CI: 0.915–0.962) for SIO.

## Discussion

The principal finding of this study is that quantitative MRI parameters reflect anaemia-related microstructural remodelling of the vertebral bone marrow in a severity-dependent manner. When referenced against normal marrow values, ADC differences were detectable at moderate anaemia, whereas FF changes reached statistical significance only in severe anaemia, suggesting that ADC may be a more sensitive indicator of compositional shifts—though longitudinal studies are needed to confirm whether these represent truly sequential phases of marrow adaptation or different thresholds of the same continuous remodelling process. In contrast, SIO did not show significant intergroup differences, suggesting limited sensitivity of two-point Dixon signal intensity ratios to detect mild-to-moderate changes in marrow fat content. Importantly, all quantitative measurements demonstrated excellent inter- and intra-reader agreement (ICC > 0.90), confirming the reproducibility and robustness of the applied ROI-based measurement approach. Collectively, these results highlight the complementary utility of ADC and FF as noninvasive biomarkers of anaemia-related stromal remodelling. To our knowledge, this is the first study to simultaneously evaluate lumbar ADC, FF, and SIO across WHO-defined anaemia severity categories in a non-malignant population, thereby addressing a significant gap in the existing literature.

From a clinical perspective, it should be emphasized that anaemia diagnosis and management are primarily based on laboratory findings and clinical evaluation rather than imaging alone. In this context, the principal clinical contribution of this study lies in providing a structured radiologic framework for interpreting incidental vertebral marrow signal alterations detected on abdominal MRI. The combined assessment of ADC and FF may facilitate recognition of imaging patterns consistent with anaemia-related marrow remodeling and provide quantitative information beyond conventional DWI signal intensity. Moreover, the pattern of decreased FF accompanied by relatively increased ADC in benign red marrow hyperplasia may differ from the lower ADC values typically associated with malignant marrow infiltration, likely reflecting differences in marrow cellularity and composition. Further comparative studies are needed to clarify the diagnostic value of these quantitative patterns in differentiating benign marrow remodeling from malignant infiltration.

The observed decrease in FF with increasing anaemia severity reflects a biological response consistent with reduced bone marrow fat content and reactive hematopoietic hyperplasia. As Hb levels decline and tissue hypoxia develops, erythropoietic activity increases, leading stromal adipose tissue to be progressively replaced by red marrow cells. Our findings align with previous studies reporting that FF reflects reductions in stromal fat content [6, 9, 22].

Another key finding of this study is the inverse association between hemoglobin levels and ADC values. ADC elevation in anemia likely reflects compositional changes within the bone marrow, including reduced marrow fat content and an increased free-water fraction associated with anemia-related marrow remodeling. This interpretation is consistent with prior work demonstrating higher ADC values in bone marrow with reduced adiposity [23–25] including conditions such as Gaucher disease and multiple myeloma [23, 24]. Conversely, Tsujikawa et al. [7] reported a positive association between hemoglobin and iliac but not lumbar ADC. Differences in field strength, fat suppression technique, and *b*-values likely contributed to signal variability in their whole-body protocol. Our use of a standardized abdominal DWI protocol at 1.5 T and the exclusion of malignancy or treatment-related marrow changes likely provided more stable diffusion measurements, resulting in a clearer negative association between Hb and lumbar ADC.

The lack of significant differences in SIO across anaemia groups deserves particular consideration. Two-point Dixon-derived OP/IP ratios represent a qualitative fat metric and are inherently more susceptible to noise, B0 inhomogeneity, and phase-cancellation artefacts compared with multi-echo Dixon-based PDFF measurements. As a result, SIO is less sensitive to gradual and physiologic stromal remodelling. Importantly, anaemia causes a continuum of mild-to-moderate reductions in marrow adiposity rather than the abrupt and extensive fat loss seen in malignant infiltration, where SIO has been shown to perform well in prior studies [6, 26]. This distinction likely explains why SIO remained nondiscriminatory even in the severe anaemia group in our cohort. Moreover, the vertebral marrow is particularly prone to field heterogeneity, further limiting the reliability of OP/IP ratios. Collectively, these factors, combined with the absence of prior studies evaluating SIO differences across WHO-defined anaemia severity categories, highlight the methodological limitations of SIO and reinforce the superior sensitivity of quantitative PDFF for detecting anaemia-related microstructural changes.

The differential associations observed between RDW-CV and RDW-SD may be related to the distinct analytical properties of these indices. RDW-CV represents a coefficient of variation derived from the standard deviation of erythrocyte size normalized to mean corpuscular volume (MCV), whereas RDW-SD reflects the absolute width of the erythrocyte volume distribution [27]. In our cohort, RDW-CV demonstrated significant correlations with bone marrow MRI parameters in unadjusted analyses, whereas RDW-SD showed significant associations only after adjustment, suggesting that its relationship with marrow microstructure may be influenced by confounding factors. Notably, the initially weak and borderline associations between RDW-SD and both ADC and FF became more robust after adjustment. For RDW-SD–ADC, statistical significance was achieved after both age and BMI adjustment, whereas for RDW-SD–FF, this effect was primarily driven by age adjustment. The limited impact of BMI on FF may be explained by the fact that BMI does not directly reflect bone marrow adiposity but rather overall body composition. In contrast, age is a primary determinant of marrow fat content, characterised by progressive fatty replacement of haematopoietic tissue, and may therefore exert a more pronounced masking effect on these associations. Overall, these observations likely reflect differences in the statistical and computational characteristics of these indices rather than differences in their ability to reflect anaemia severity.

This study has several limitations. The retrospective design and relatively small sample size may restrict the generalizability of the findings to broader populations. Moreover, the cross-sectional design precludes conclusions regarding the temporal evolution of marrow remodelling across anemia severity stages, which would require longitudinal studies. The absence of histopathological confirmation limits the ability to directly correlate MRI-derived microstructural measures with cellular alterations in the bone marrow. Additionally, all examinations were performed using a single magnetic field strength (1.5 T), preventing assessment of potential variations attributable to different scanner platforms or sequence parameters. Furthermore, ROI placement was limited to a single lumbar vertebral level, which may not fully represent global bone marrow status. Future multicentre, prospective studies with larger cohorts, multi-level sampling strategies, and advanced techniques such as histogram analysis or artificial intelligence–based tissue characterization may enable a more comprehensive evaluation of anemia-related microstructural alterations.

In conclusion, this study demonstrates that anaemia-related alterations in bone marrow microstructure can be assessed using quantitative MRI parameters. ADC and FF measurements may serve as potential noninvasive biomarkers that reflect the interplay between hematopoietic activity and stromal fat balance, thereby providing insight into the microstructural and compositional changes associated with varying degrees of anaemia severity.

## Data Availability

The datasets generated and/or analyzed during the current study are not publicly available due to patient privacy and institutional restrictions but are available from the corresponding author on reasonable request.
